# Skin autofluorescence and the complexity of complications in patients with type 2 diabetes mellitus: a cross-sectional study

**DOI:** 10.1186/s12902-021-00725-6

**Published:** 2021-04-01

**Authors:** Xian Wang, Xingwang Zhao, Tingting Lian, Juanjin Wei, Wanxu Yue, Senwei Zhang, Qiu Chen

**Affiliations:** 1 School of Clinical Medicine, Hospital of Chengdu University of Traditional Chinese Medicine, Chengdu, China; 2 Department of Endocrinology, Jianyang Traditional Chinese Medicine Hospital, Chengdu, China; 3 Department of Endocrinology, General Hospital of Southern Theater Command, Guangzhou, China; 4 Department of Endocrinology, Hospital of Chengdu University of Traditional Chinese Medicine, Chengdu, 610072 China

**Keywords:** Skin autofluorescence, T2DM complications, Advanced glycation end products

## Abstract

**Background:**

The accumulation of advanced glycation end products (AGEs) occurring in skin tissues can be measured as skin autofluorescence (SAF). Here, we assessed the correlation between SAF values and the complexity and severity of type 2 diabetes mellitus (T2DM) complications.

**Methods:**

The basic clinical information of 825 patients with T2DM was collected through an electronic system, and SAF was measured by adapting a DM-Scan, a non-invasive optical signal detector. Diabetic complications were diagnosed based on clinical criteria by experienced doctors. Linear regression analysis was used to evaluate the independent determinants of SAF, and multiple logistic regression analysis was performed to assess independent determinants that influence the severity of the complications.

**Results:**

SAF was significantly associated with the complexity of T2DM complications. Similarly, independent relationships between SAF and age (*β* = 0.389, *P* <  0.001), sex (*β* = − 2.221, *P* = 0.004), 2-h C-peptide (*β* = − 0.182, *P* = 0.017), aminotransferase (ALT, *β* = − 0.158, *P* = 0.041), blood creatinine (BCr, *β* = 0.206, *P* = 0.009), and fatty liver (*β* = 0.161, *P* = 0.026) were observed. With the increasing number of complications, the SAF values increased significantly after adjusting for related risk factors. The SAF values correlated with diabetic retinopathy, diabetic kidney diseases, cardiovascular disease, and diabetic peripheral neuropathy when compared with patients without any T2DM-associated complications. Moreover, the AGE-based diabetic complication risk score for each complication demonstrated a relationship with the presence or absence of certain complications.

**Conclusion:**

SAF is an independent marker for diabetic retinopathy, diabetic kidney diseases, cardiovascular disease, and diabetic peripheral neuropathy, and it is also a predictor of the complexity of T2DM complications. Moreover, the diabetic complication risk score is capable of predicting the risk of diabetic complications in patients with T2DM.

## Background

Due to advances in healthcare, especially the widespread application of rapid serum glucose screening, the occurrence of complications associated with diabetes has significantly decreased [1]. However, diabetes and its complications still rank as one of the most common causes of death and quality-of-life deterioration due to disease progression [2]. Although long-term survival can be achieved in patients with diabetes complications, the majority of these patients live poor-quality lives and suffer further disease progression and even death [3]. Importantly, although serum glucose levels can be easily monitored via point-of-care tests, there are no appropriate clinical biomarkers for the early diagnosis and/or detection of diabetes complications [4].

Among all the known factors, hyperglycaemia contributes significantly to diabetes complications, especially vascular complications [5]. Persistent hyperglycaemia in patients with diabetes, accompanied by hyperlipidaemia, oxidative/carbonyl stress and decreased renal function, leads to the accumulation of advanced glycation end products (AGEs), which is considered to be one of the major pathogenetic mechanisms resulting in end-organ damage in these patients [6]. In principle, AGE-related cross-linking and receptor-mediated cellular activation should affect multiple organ systems, presenting as a loss of elasticity of the vascular wall and cellular inflammation, which would result in microvascular and macrovascular complications [7]. Consequently, AGEs could serve as a potential biomarker for complications related to diabetes.

The accumulation of AGEs often occurs in skin tissues over time due to their low turnover rate. As such, the detection of AGEs in skin biopsies has been used as a predictor of long-term diabetic complications in a prior study employing a large cohort, even after adjusting for haemoglobin A1c (HbA1c) levels [8]. However, the invasive and time-intensive nature of skin biopsies has limited the application of this method for at-risk outpatients. Recently, a non-invasive method of measuring skin autofluorescence (SAF) using an autofluorescence reader was developed [9]. Prior studies have shown that SAF is strongly correlated with specific AGEs in skin biopsies and is related to the progression of vascular complications. However, most of these studies only enrolled Caucasian patients, and racial differences have been reported for SAF. In addition, the association of SAF and the severity and complexity of complications associated with diabetes has only seen limited investigation.

In this study, we collected basic clinical information, measured the SAF of patients diagnosed with type 2 diabetes mellitus (T2DM), and further analysed the relationship between the SAF value and diabetic complications to determine the role of the SAF value in the severity and complexity of diabetic complications.

## Methods

### Study population

A total of 825 patients with T2DM aged 18 to 80 years old who were treated at the Hospital of Chengdu University of Traditional Chinese Medicine from January 2015 to May 2019 were enrolled in this study. The diagnosis of T2DM was based on the diagnosis and classification of diabetes mellitus by the American Diabetes Association [10]. The exclusion criteria were as follows: 1) other special types of diabetes, such as type I diabetes, mitochondrial gene mutation diabetes, gestational diabetes, and adult-type autoimmune diseases in young people; 2) malignant digestive tract tumours; 3) various acute and chronic infections; 4) mental and neurological diseases; and 5) liver and kidney failure and severe heart disease. The study was approved by the ethics committees at the Hospital of Chengdu University of Traditional Chinese Medicine (No. 2017KL-033).

### Clinical data collection

All of the patients had full medical history records in the electronic system, which included all test results. The general indicators for these patients included age, sex, body mass index (BMI), fasting blood glucose (FBG), 2-h postprandial blood glucose (2-h PBG), HbA1c, fasting C-peptide and 2-h standardized postprandial C-peptide (2-h C-peptide). In addition, total cholesterol (Tch), triglycerides (TGs), high-density lipoprotein cholesterol (HDL-C), low-density lipoprotein cholesterol (LDL-C), alanine aminotransferase (ALT), aspartate aminotransferase (AST), *γ*-glutamyltransferase (GGT), blood creatinine (BCr), blood uric acid (BUA), blood urea nitrogen (BUN), cystatin-C (Cys-C), and homocysteine (HCY) were detected to estimate blood lipids, liver function, and kidney function. The Cockcroft-Gault equation was used to determine the estimated glomerular filtration rate (eGFR) [11]. Smoking/drinking behaviour was defined as smoking/drinking or never. Hypertension was defined as either 1) using anti-hypertensive medication or 2) systolic blood pressure (SBP) ≥ 140 mmHg or diastolic blood pressure (DBP) ≥ 90 mmHg. The diagnosis of fatty liver was primarily confirmed via ultrasonography by an experienced physician.

### SAF assessment and diabetic complication risk score

Upon excitation at 370 nm, AGEs have a characteristic fluorescence spectrum at 440 nm. Skin long wavelength fluorescence ~ excitation (ex)/emission (em) 370/440 nm was introduced as a surrogate marker for AGE formation and has since been widely used in both clinical and experimental studies of diabetes [12, 13]. In this study, skin AGEs were assessed by an autofluorescence reader (Hefei Institutes of Physical Science, Chinese Academy of Sciences) following a 2–3-min measurement of the left volar forearm [14–16]. In short, an excitation light source with a peak wavelength of 370 nm was used to excite the AGEs in the skin that have fluorescence properties in a frequency range of 420–600 nm, and a broadband light source in a frequency range of 420–600 nm illuminated the skin to measure tissue absorption and scattering. Emission light, including reflected light (diffuse reflectance light) and fluorescence from the skin, was measured with a spectrometer in the 300–600-nm range through a 3 × 1 fibre bundle. AGE-related autofluorescence was determined from the fluorescence and reflected light. The final values were relative (the ratio of fluorescence intensity and diffuse reflectance light intensity), so there was no uniform range, and the range of AGE values we used was 0–150.

The diabetic complication risk score was also given by an autofluorescence reader with a built-in algorithm (Hefei Institutes of Physical Science, Chinese Academy of Sciences). The device has been certified by the CFDA (China Food and Drug Administration) and CE. The risk score is a prediction model that utilizes an algorithm to combine various biological markers of diabetic complications. In detail, many studies have shown that AGEs can cause neuropathy, nephropathy, retinopathy, and cardiovascular complications through both receptor and non-receptor pathways [17]. The higher the AGEs of the individual, the higher is the risk of complications. Second, the four questions in the embedded software regarding the clinical symptoms are as follows: numbness of the hands and feet corresponding to neuropathy [18], blurred vision corresponding to retinopathy [19], hypertension and hyperlipidaemia corresponding to cardiovascular disease [20], and body oedema corresponding to kidney disease [21]. Third, some common tissue spectral features were used to evaluate complications, for example, the ratio of reflectance at 390 and 360 nm, deviation of UV reflectance from a straight line, ratio of reflectance at 470 and 500 nm, ratio of emission at 470 and 500 nm, and ratio of emission at 470 and 570 nm. Multiple logistic regression analysis was used to determine the β coefficient of the AGE value, questionnaire and characteristic spectrum. Finally, the total score range was converted to 0–100.

All measurements were performed at room temperature in a semi-dark environment by trained nurses. For every patient, the fluorescence of the skin was measured 3 times on the volar side of the arm on normal skin without visible vessels, scars, lichenification, or other skin abnormalities.

### Definition of T2DM complications

Major T2DM complications in this study included diabetic retinopathy (R), diabetic kidney disease (K), cardiovascular disease (C) and diabetic peripheral neuropathy (N). Diabetic retinopathy was diagnosed with five grades by professional ophthalmologists in accordance with the Global Diabetic Retinopathy Project G 2002 [22], including 0 (no), 1 (mild), 2 (moderate), 3 (severe), and 4 (proliferative). In this study, as a result of the sample size, we divided the classification into three groups for better comparison: 0, ≤ 2, and ≥ 3. Diabetic kidney disease was diagnosed when UACR ≥30 mg/g/Cr and/or eGFR ≤30 ml/min/1.73 m^2^ according to the Classification of Diabetic Nephropathy 2014 [23]. Cardiovascular disease was defined as a history of ischaemic heart disease and/or a history of percutaneous coronary intervention or coronary bypass surgery according to International Classification of Diseases (ICD) codes I20–25. Diabetic peripheral neuropathy includes 1) neuropathy at or after the diagnosis of diabetes; 2) clinical signs and symptoms consistent with the performance of DPN; 3) clinical symptoms (pain, numbness, etc.); or 4) one abnormality of the five examinations (ankle reflexes, discrimination, vibration perception, pinprick sensation, or 10-g monofilament) with symptoms or two without symptoms.

### Statistical analysis

Continuous variables that conformed to a normal distribution are expressed as the mean ± standard deviation, while non-normally distributed variables are expressed as the median with the interquartile range. ANOVA and the Kruskal-Wallis/Mann-Whitney tests were used for comparisons among groups. Frequencies were used to describe the distribution of categorical variables. Linear regression analysis was used to evaluate the independent determinants of SAF, and then multiple logistic regression analysis was performed to assess the independent determinants that influence the severity of the complications. A two-tailed *P*-value of less than 0.05 was defined as statistically significant. All of the statistical analyses were performed using SPSS 23.0 statistical software (IBM, USA).

## Results

### Clinical characteristics

A total of 825 patients with T2DM were included in this study. All of the participants were classified into five groups based on their number of complications (74 patients in group 0, 133 in group 1, 201 in group 2, 275 in group 3, and 142 in group 4). From groups 0 to 4, the median age increased from 44 to 68 (*P* <  0.001). Similarly, T2DM duration, categorized by years as 0–5, 10–15, 15–20, and > 20, was significantly different among the five groups (*P* <  0.05). However, no significant difference was apparent for years 5–10 (*P* = 0.255). Additionally, there was no difference in the history of smoking or drinking among the five groups. For the laboratory tests, Tch (*P* = 0.013), TGs (*P* <  0.001), ALT (*P* = 0.028), BCr (*P* <  0.001), BUA (*P* <  0.001), Cys-C (*P* = 0.025), and eGFR (*P* <  0.001) were significantly different among the groups, but there was no significant difference in FBG, 2-h PBG, fasting C-peptide, 2-h C-peptide, HbA1c, HDL-C, LDL-C, AST, GGT, BUN, or HCY. Expressly, the SAF levels were significantly different among the five groups, and the median SAF value increased from group 0 to group 4: 76.35 (95% CI: 71.45–80.15) in group 0; 79.6 (72.45–89.9) in group 1; 84.7 (77.05–94.7) in group 2; 86.6 (76.7–96.2) in group 3; and 91.85 (81.63–102.5) in group 4 (*P* <  0.001). There were also significant differences among the four types of risk scores associated with the complications (Table 1).

**Table 1:** Clinical characteristics of different number of diabetic complications

	Number of Diabetic Complications					***P*** value
	0 (***n*** = 74)	1 (***n*** = 133)	2 (***n*** = 201)	3 (***n*** = 275)	4 (***n*** = 142)	
**Age (years)**	44 (36–50)	52 (46–61)	62 (52–69)	63 (54–71)	68 (55–75.25)	< 0.001^***^
**Female (n)**	32 (43.24%)	75 (55.97%)	100 (49.75%)	158 (57.66%)	80 (56.34%)	0.374
**BMI (kg/m**^**2**^**)**	25.01 ± 3.34	23.89 ± 3.33	24.53 ± 3.58	24.08 ± 3.30	24.58 ± 3.54	0.156
**Smoke (n)**	30 (40.54%)	51 (38.06%)	63 (31.34%)	88 (32%)	50 (35.21%)	0.774
**Drink (n)**	21 (28.38%)	42 (31.34%)	61 (30.35%)	84 (30.55%)	33 (23.24%)	0.77
**Duration (years)**						
0–5	51 (70.83%)	77 (57.46%)	64 (31.84%)	87 (31.64%)	21 (14.79%)	< 0.001^***^
5–10	16 (21.62%)	52 (38.8%)	66 (32.84%)	77 (28%)	37 (26.06%)	0.255
10–15	7 (9.46%)	18 (13.43%)	32 (15.92%)	55 (20%)	41 (28.87%)	0.019^*^
15–20	0 (0%)	5 (3.7%)	18 (9%)	29 (10.55%)	18 (12.68%)	0.009^**^
> 20	0 (0%)	1 (0.7%)	20 (1%)	27 (9.82%)	25 (17.61%)	< 0.001^***^
**SAFs (AU)**	76.35 (71.45–80.15)	79.6 (72.45–89.9)	84.7 (77.05–94.7)	86.6 (76.7–96.2)	91.85 (81.63–102.5)	< 0.001^***^
N value (score)	64.3 (59.35–73.4)	65.8 (59.45–76.35)	74.1 (64.6–81.55)	74.7 (64.8–82)	77.8 (68.38–85.73)	< 0.001^***^
R value (score)	62.35 (57.58–68.88)	66.3 (57.93–77.23)	74.1 (63.65–80.7)	75.1 (63.3–79.9)	78 (68.28–84.33)	< 0.001^***^
K value (score)	62.7 (58.03–64.8)	64.5 (56.05–71.2)	66.1 (62.05–77.25)	67.1 (62–76.9)	74 (64.98–85.08)	< 0.001^***^
C value (score)	71.8 (64.58–79.38)	74.2 (64.5–81.8)	78.8 (67.6–83.55)	79.5 (67–85.3)	83.6 (74.38–89.4)	< 0.001^***^
**Lab tests**						
FBG (μmol/L)	7.7 (6.21–10.14)	7.33 (6.19–9.66)	7.92 (6.3–11.46)	7.9 (6.17–10.97)	8.65 (6.31–11.39)	0.696
2-h PBG (mmol/L)	16.91 ± 5.67	16.63 ± 5.09	18.04 ± 4.29	18.21 ± 4.83	18.52 ± 5.66	0.078
Fasting C-peptide (ng/mL)	0.8 (0.67–1)	0.67 (0.5–0.87)	0.7 (0.5–0.9)	0.66 (0.48–0.9)	0.79 (0.51–0.98)	0.091
2-h C-peptide (ng/mL)	1.77 (1.35–2.64)	1.77 (1.25–2.46)	1.61 (1.22–2.25)	1.68 (1.1–2.34)	1.47 (0.12–2.05)	0.09
HbA1c (%)	8.3 (6.1–11)	7.65 (6.2–10.55)	8.0 (6.5–10.7)	7.8 (6.3–10.2)	8 (6.65–10.55)	0.164
Tch (mmol/L)	4.47 (3.88–5.47)	4.45 (3.89–5.3)	4.36 (3.68–5.14)	4.3 (3.64–4.94)	4.49 (3.55–5.35)	0.013^*^
TGs (mmol/L)	2.1 (1.37–3.78)	1.66 (1.25–2.52)	1.52 (1.11–2.39)	1.64 (1.06–2.44)	1.74 (1.24–2.68)	< 0.001^***^
HDL-C (mmol/L)	0.97 (0.84–1.13)	1.04 (0.9–1.22)	1.07 (0.88–1.32)	1.04 (0.88–1.2)	1.02 (0.89–1.2)	0.473
LDL-C (mmol/L)	2.69 (2.09–3.21)	2.66 (2.21–3.12)	2.59 (2.01–3.13)	2.55 (2.05–3.06)	2.56 (1.88–3.15)	0.813
ALT (IU/L)	24 (15.5–41)	23 (16–38.75)	20 (12–35)	20 (15–32)	19 (14–28.75)	0.028^*^
AST (IU/L)	19 (15.5–26)	20 (16–27)	19 (16–26.75)	20 (15–25)	19 (16–24)	0.727
GGT IU/L)	27 (19–47)	25 (18–43.25)	24 (17–44)	25 (17–37.25)	26 (18–37.5)	0.824
BCr (*μ*mol/L)	58 (46.75–65.8)	59.6 (48.28–69.33)	62.05 (52.6–78.08)	63.1 (52.6–78.4)	77.8 (60.8–119)	< 0.001^***^
BUA (*μ*mol/L)	348 (274.5–395.5)	309 (257–379)	325 (275–386.8)	328 (281–382.5)	383 (315.5–443)	< 0.001^***^
BUN (mmol/L)	4.74 (4.12–6.07)	4.9 (3.83–6.37)	5.07 (4.09–6.37)	5.5 (4.44–7.26)	7.22 (5.36–9.63)	0.382
Cys-C (*μ*mol/L)	0.74 (0.64–0.86)	0.8 (0.68–0.96)	0.96 (0.78–1.21)	0.94 (0.79–1.22)	1.22 (0.91–1.78)	0.025^*^
eGFR (mL/min)	129.9 (106.2–156.1)	112.4 (96.44–132.3)	98.28 (81.16–111)	96.82 (77.52–107.7)	78.95 (46.97–99.03)	< 0.001^***^
HCY (*μ*mol/L)	9.22 (6.35–11.8)	9.25 (7.67–12.31)	9.54 (7.91–12.89)	10.24 (8.37–13.49)	12.3 (9.97–16.51)	0.585
**Hypertension (n)**	15 (20.27%)	31 (23.13%)	75 (37.31%)	130 (47.27%)	97 (68.31%)	< 0.001^***^
**Fatty liver (n)**	44 (59.46%)	36 (53%)	24 (63%)	31 (71%)	10 (38%)	< 0.001^***^

Data are shown as the median (range), mean ± SD or number (percentage). ^*^ *P* < 0.05, ^**^ *P* < 0.01, ^***^ *P* < 0.001, among groups with different number of diabetic complications

### Correlation of the SAF value with other variables

Univariate linear regression showed an association between SAF and age (*R* = 0.479, *P* <  0.001), sex (*R* = − 0.070, *P* = 0.045), smoking (*R* = 0.140, *P* <  0.001), drinking (*R* = 0.151, *P* <  0.001), T2DM duration (*R* = 0.260, *P* <  0.001), hypertension (*R* = 0.134, *P* <  0.001), and fatty liver (*R* = 0.103, *P* = 0.021) as well as the levels of 2-h PBG (*R* = 0.141, *P* = 0.007), 2-h C-peptide (*R* = − 0.140, *P* = 0.007), TGs (*R* = − 0.136, *P* <  0.001), ALT (*R* = − 0.143, *P* <  0.001), BCr (*R* = 0.387, *P* <  0.001), BUA (*R* = 0,178, *P* <  0.001), Cys-C (*R* = 0.243, *P* <  0.001), and eGFR (*R* = − 0.301, *P* <  0.001). However, BMI, FBG, fasting C-peptide, HbA1c, Tch, HDL-C, LDL-C, AST, GGT, BUN, and HCY showed no relationship with SAF levels. Moreover, multivariate analysis indicated that age (*β* = 0.389, *P* <  0.001), sex (*β* = − 2.221, *P* = 0.004), 2-h C-peptide (*β* = − 0.182, *P* = 0.017), ALT (*β* = − 0.158, *P* = 0.041), BCr (*β* = 0.206, *P* = 0.009), and fatty liver (*β* = 0.161, *P* = 0.026) were independent determinants of SAF (Table 2).

**Table 2:** Relative risk factors of SAF in T2DM

	Univariate analysis		Multivariate analysis	
	***R***	***P*** value	***β***	***P*** value
**Age (years)**	0.479	< 0.001^***^	0.389	< 0.001^***^
**Female (n)**	- 0.070	0.045^*^	- 2.221	0.004^**^
**BMI (kg/m**^**2**^**)**	- 0.052	0.139		
**Smoke (n)**	0.140	< 0.001^***^	–	NI
**Drink (n)**	0.151	< 0.001^***^	–	NI
**Duration (years)**	0.260	< 0.001^***^	–	NI
**Lab tests**				
FBG (*μ*mol/L)	0.010	0.769		
2-h PBG (mmol/L)	0.141	0.007^**^	–	NI
Fasting C-peptide (ng/mL)	- 0.016	0.763		
2-h C-peptide (ng/mL)	- 0.140	0.007^**^	- 0.182	0.017^*^
HbA1c (%)	- 0.042	0.287		
Tch (mmol/L)	- 0.240	0.5		
TGs (mmol/L)	- 0.136	< 0.001^***^	–	NI
HDL-C (mmol/L)	- 0.008	0.823		
LDL-C (mmol/L)	- 0.001	0.978		
ALT (IU/L)	- 0.143	< 0.001^***^	- 0.158	0.041^*^
AST (IU/L)	- 0.058	0.095		
GGT IU/L)	- 0.009	0.799		
BCr (*μ*mol/L)	0.387	< 0.001^***^	0.206	0.009^**^
BUA (*μ*mol/L)	0.178	< 0.001^***^	–	NI
BUN (mmol/L)	0.047	0.179		
Cys-C (*μ*mol/L)	0.243	< 0.001^***^	–	NI
eGFR (mL/min)	- 0.301	< 0.001^***^	–	NI
HCY (*μ*mol/L)	0.039	0.287		
**Hypertension (n)**	0.134	< 0.001^***^	–	NI
**Fatty liver (n)**	0.103	0.021^*^	0.161	0.026^*^

Linear regression analysis was performed to determine the predictors of SAF. *P < 0.05, **P < 0.01, ***P < 0.001. The significant (*P* < 0.05) variables in the univariate analysis were selected for multivariate regression analysis. NI: no significance (not an independent determinant)

### Association of complications with SAF in patients with T2DM

**Fig. 1:**
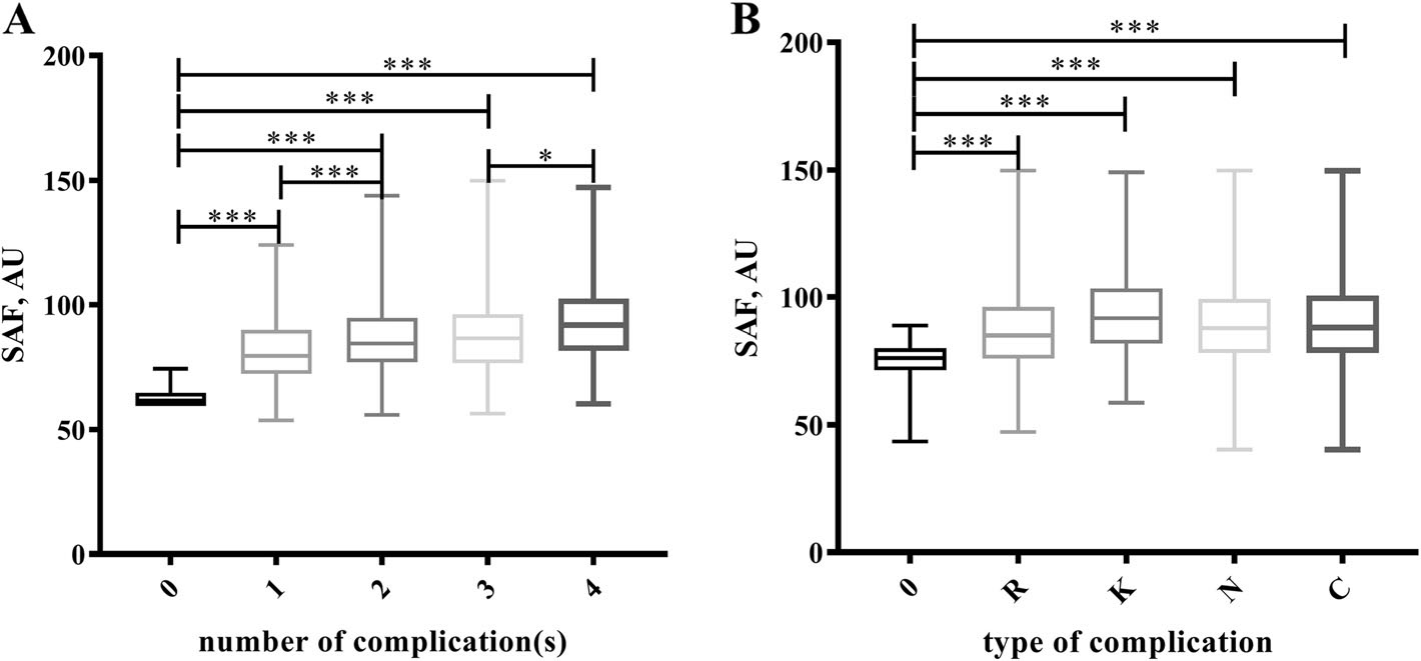
Relationship between SAF and T2DM complications. **a** Relationship between SAF and the number of T2DM complications; **b** Relationship between SAF and a certain type of T2DM complication. * *P* <  0.05, *** *P* <  0.001

ANOVA tests were performed to compare the SAF values of enrolled patients with different numbers of complications, and the results showed that the SAF values of patients with 1, 2, 3, and 4 complications were significantly increased compared with those with 0 complications. Moreover, there were significant differences between the patients with 1 vs. 2 and 3 vs. 4 complications (*P* <  0.05), while there was no significant difference between 2 and 3 complications, as shown in Fig. 1a. In addition, we compared the SAF values of no complications and each individual complication (0 vs. R, diabetic retinopathy; 0 vs. K, diabetic kidney diseases; 0 vs. N, diabetic peripheral neuropathy; 0 vs. C, cardiovascular disease), and the *P*-values among the four groups were <  0.001, as shown in Fig. 1b. Logistic regression in the unadjusted model indicated that the SAF value had a significant association with the number of complications in reference to no complications (*P* <  0.001). After adjusting for age, sex, and BMI in model 2, the *P*-value for the trend was 0.007. Similarly, we calculated the variables, including FBG, HbA1c, C-peptide, history of smoking, drinking, and hypertension, in model 3, and this showed that the P-value for the trend was 0.023; however, after adding TGs, BUA, BCr, and eGFR to model 4, there was no significant association (*P* = 0.129), as shown in Table 3. In addition, the correlation between SAF and certain types of T2DM complications was analysed after adjusting for the relative risk factors, as presented in Table 4. In the unadjusted model, SAF had a significant relationship with only diabetic kidney disease, diabetic cardiovascular disease, and diabetic neuropathy (*P* <  0.001), but SAF demonstrated no relation with diabetic retinopathy. Nevertheless, after adjusting for the associated variables in model 2, there was no significant association between SAF and diabetic cardiovascular disease. Ultimately, diabetic kidney disease (*P =* 0.007) and diabetic neuropathy (*P* <  0.001) showed a statistically significant connection with SAF.

**Table 3:** The correlation between SAF and the number of T2DM complications

	0	1	2	3	4	*P*
Model 1	1 (Ref)	1.18 (0.71–1.68)	1.25 (1.13–1.77)	1.35 (1.20–1.90)	1.89 (1.45–2.24)	< 0.001^***^
Model 2	1 (Ref)	1.28 (1.09–1.74)	1.32 (1.17–1.82)	1.42 (1.15–2.17)	2.01 (1.75–2.85)	0.007^**^
Model 3	1 (Ref)	2.01 (0.61–3.70)	2.25 (1.53–2.79)	2.51 (1.60–2.72)	2.89 (1.45–2.26)	0.023^*^
Model 4	1 (Ref)	1.81 (0.62–2.12)	2.15 (0.81–3.09)	2.21 (1.72–2.93)	3.09 (1.89–3.57)	0.129

Model 1: Not adjusted

Model 2: AGE, Age, Sex, BMI

Model 3: AGE, Age, Sex, BMI, FBG, HbA1c, C-pep, Smoking, Drinking, Hypertension

Model 4: AGE, Age, Sex, BMI, FBG, HbA1c, C-pep, Smoking, Drinking, Hypertension, TGs, BUA, Cr, eGFR

*P < 0.05, **P < 0.01, ***P < 0.001

**Table 4:** The correlation between SAF and certain types of T2DM complications

	R		K		C		N	
	OR (95%CI)	*P*	OR (95%CI)	*P*	OR (95%CI)	*P*	OR (95%CI)	*P*
Model 1	1.004 (0.994–1.013)	0.453	1.044 (1.034–1.054)	< 0.001^***^	1.018 (1.009–1.027)	< 0.001^***^	1.056 (1.042–1.071)	< 0.001^***^
Model 2			1.032 (1.021–1.043)	< 0.001^***^	0.995 (0.985–1.005)	0.33	1.039 (1.024–1.054)	< 0.001^***^
Model 3			1.034 (1.011–1.058)	0.004			1.085 (1.046–1.126)	< 0.001^***^
Model 4			1.046 (1.013–1.080)	0.007			1.088 (1.044–1.135)	< 0.001^***^

Model 1: Not adjusted

Model 2: AGE, Age, Sex, BMI

Model 3: AGE, Age, Sex, BMI, FBG, HbA1c, C-pep, Smoking, Drinking, Hypertension

Model 4: AGE, Age, Sex, BMI, FBG, HbA1c, C-pep, Smoking, Drinking, Hypertension, TGs, BUA, Cr, eGFR

*P < 0.05, **P < 0.01, ***P < 0.001

### Assessment of the risk score for each complication

**Fig. 2:**
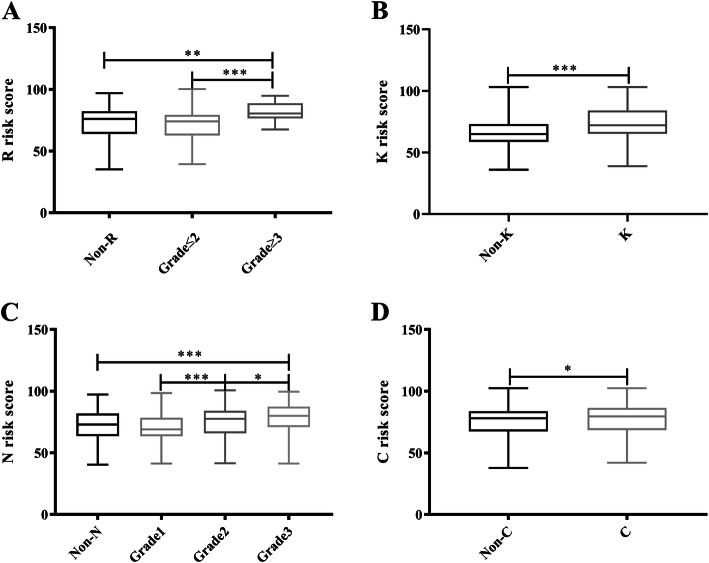
Correlation between diabetic complication risk score and certain T2DM complications. **a** R risk score in diabetic retinopathy; **b** K risk score in diabetic kidney disease; **c** N risk score in diabetic peripheral neuropathy; **d** C risk score in cardiovascular disease. * *P* <  0.05; ** *P* <  0.01; *** *P* <  0.001

The SAF test reported four risk scores for corresponding complications, and the evaluation effect between each complication and no complications was calculated via Mann-Whitney and ANOVA tests (Fig. 2). For diabetic retinopathy, there were significant differences between non-diabetic retinopathy and retinopathy grade ≥ 3 (*P* = 0.003) as well as between grade ≤ 2 and ≥ 3 (*P* <  0.001), but there was no difference between non-diabetic retinopathy and grade ≤ 2 (*P* = 0.073). Additionally, there was a difference between non-diabetic kidney disease and diabetic kidney disease (*P* <  0.001), and the same result was observed in diabetic cardiovascular disease (*P* = 0.037). It should be noted that there were significant differences between grade 1 vs. grade 2 (*P* <  0.001) and grade 2 vs. grade 3 (*P* = 0.01) with regard to diabetic peripheral neuropathy, while only grade 3 diabetic peripheral neuropathy showed a significant difference compared with non-diabetic peripheral neuropathy (*P* <  0.001).

## Discussion

Previous studies have shown that there was a significant difference in the levels of SAF between patients with and without T2DM [24, 25]. Moreover, the levels of SAF were also closely associated with the occurrence of diabetic complications [25–35]. The results of our study were consistent with the above research. In addition, this study also discussed in detail for the first time that there was a close relationship between SAF and the number of complications in patients with T2DM.

Similar to prior studies, certain factors might have some influence on SAF levels, including age [24, 32, 33, 36, 37], female sex [36, 37], BCr [33], and C-peptide [33]. However, our study also assessed additional factors, including smoking history, BMI, and diabetes duration. Smoking history was not an influential factor in our study, probably because smoking was defined as a two-category variable, whereas prior studies analysed smoking based on pack-years as a quantitative variable [24]. In terms of BMI, we did not find any significant difference among the five groups at baseline, leading us to speculate that the larger sample size employed in our study was the main reason for this discrepancy. Apart from this, prior studies have shown that there is a strong relationship between the duration of diabetes and SAF [33, 36, 37]. However, this current study divided this duration into five grades, and the sample size varied in each grade. As such, we did not identify this association in this study.

In the present study, we found two other independent risk factors that were associated with SAF: ALT and fatty liver. Based on these findings, we proposed that there is a close relationship between liver diseases and SAF. Prior research has revealed part of a potential related mechanism wherein the inactivation of hepatic AMP-activated protein kinase by fructose-mediated AGEs leads to an increase in methylglyoxal flux, subsequently perpetuating lipogenesis and fatty liver [38].

A positive correlation between SAF levels and the number of complications is presented in this study, and this significant correlation still existed after adjusting for age, sex, BMI, FBG, HbA1c, C-pep, smoking, drinking, and hypertension (model 3). However, after also adjusting for TGs, BUA, BCr, and eGFR, this significant difference was absent. This was likely because SAF itself was greatly affected by renal function, as it was regulated through the mechanistic pathway of kidney podocyte AGE-R1 activation and exposed to an environment of excessive AGEs [39]. Furthermore, as the number of diabetic complications increased, the SAF value also increased. However, we did not find any significant difference in the complications between groups 2 and 3, probably because the sample size was not large enough. In the future, a larger sample size will be needed to verify this conclusion.

For this study, we adapted a new risk assessment system for each diabetic complication, which has not been previously detailed. Based on these values, statistical significance was apparent for diabetic nephropathy, diabetic retinopathy, diabetic cardiovascular diseases, and diabetic peripheral neuropathy above grade 2. Moreover, no significant difference was observed in grade 1 diabetic peripheral neuropathy, which may be due to the asymptomatic distal polyneuropathy stages N0 (no polyneuropathy) and N1 (asymptomatic polyneuropathy), and current monitoring is insufficient to distinguish between the two grades.

Although previous studies have found a correlation between SAF and diabetic complications, this study was the first to examine the relationship between SAF and the number of T2DM complications in detail. Additionally, the sample size of this study was larger than that of previous studies. Importantly, liver disease was taken as a risk factor in this study, and fatty liver was found to be closely related to SAF, providing a foundation for further research on the relationship between SAF and hepatic complications in patients with T2DM. Furthermore, a separate risk assessment system was used for each T2DM complication that had not been previously published. Moreover, we observed significant differences between patients with and without certain complications. Therefore, we hypothesized that it should be possible to predict the risk for patients with T2DM of suffering each kind of complication in the future.

## Limitations

The research was designed based on cross-sectional data, which might have resulted in some bias during the analysis. Consequently, prospective studies will be needed to validate the role of SAF in predicting diabetic complications in the future. There was no independent relationship between SAF and blood glucose or glycated haemoglobin in this study; however, prior studies have shown that SAF is positively correlated with the average value of long-term glycated haemoglobin [30]. This difference might have been influenced by skin pigmentation due to the degree of sun exposure. Moreover, the SAF value might be different from AGEs in the serum or body tissues among different human races or people from different regions. Finally, we only identified patients with diabetic cardiovascular complications based on medical history, but there were some patients who did not receive cardiovascular examinations or were not diagnosed clinically. Therefore, asymptomatic cardiovascular disease may have been overlooked in this study.

## Conclusion

In conclusion, SAF is an independent predictor of T2DM complications, including diabetic retinopathy, diabetic kidney disease, diabetic cardiovascular disease and diabetic peripheral neuropathy. Additionally, as the number of complications increases, the SAF value also increases. The risk score based on AGEs for each complication can also independently predict the occurrence of the complication.

## Data Availability

The datasets used and/or analysed during the current study are available from the corresponding author on reasonable request.

## Abbreviations

T2DM: Type 2 diabetes mellitus
AGEs: Advanced glycation end products
SAF: Skin autofluorescence
R: Diabetic retinopathy
K: Diabetic kidney diseases
N: Diabetic peripheral neuropathy
C: Cardiovascular disease
BMI: Body mass index
Lab: Laboratory
FBG: Fasting blood glucose
2-h PBG: 2-h postprandial blood glucose
2-h C-peptide: 2-h standardized postprandial C-peptide
HbA1c: Haemoglobin A1c
Tch: Total cholesterol
TGs: Triglycerides
HDL-C: High-density lipoprotein cholesterol
LDL-C: Low-density lipoprotein cholesterol
ALT: Alanine aminotransferase
AST: Aspartate aminotransferase
GGT: γ-glutamyltransferase
BCr: Blood creatinine
BUA: Blood uric acid
BUN: Blood urea nitrogen
Cys-C: Cystatin-C
HCY: Homocysteinel
